# Oral Glucoraphanin and Curcumin Supplements Modulate Key Cytoprotective Enzymes in the Skin of Healthy Human Subjects: A Randomized Trial

**DOI:** 10.3390/metabo15060360

**Published:** 2025-05-29

**Authors:** Anna L. Chien, Hua Liu, Saleh Rachidi, Jessica L. Feig, Ruizhi Wang, Kristina L. Wade, Katherine K. Stephenson, Aysegul Sevim Kecici, Jed W. Fahey, Sewon Kang

**Affiliations:** 1 Department of Dermatology, Johns Hopkins School of Medicine, Baltimore, MD 21287, USA; 2 Department of Pharmacology & Molecular Sciences, Johns Hopkins School of Medicine, Baltimore, MD 21205, USA; hliu8@jhmi.edu (H.L.);; 3 Department of Pediatrics, Johns Hopkins School of Medicine, Baltimore, MD 21287, USA; 4 Department of Genetic Medicine, Johns Hopkins School of Medicine, Baltimore, MD 21205, USA; 5 Department of International Health, Bloomberg School of Public Health, Johns Hopkins University, Baltimore, MD 21205, USA; 6 Department of Medicine, Johns Hopkins School of Medicine, Baltimore, MD 21205, USA; 7 Department of Psychiatry & Behavioral Sciences, Johns Hopkins School of Medicine, Baltimore, MD 21287, USA; 8 Institute of Medicine, University of Maine, Orono, ME 04469, USA

**Keywords:** broccoli, cancer, erythema, phytochemical, sulforaphane, clinical

## Abstract

**Background/Objectives:** Oxidative stress plays a pivotal role in skin aging and carcinogenesis. Phytochemicals such as sulforaphane (SF, from broccoli sprouts or seeds) or curcumin (CUR, from turmeric) can be highly protective against this stress. They each induce a suite of cytoprotective and antioxidant enzymes that are coordinately transcribed via the Keap1-Nrf2-ARE pathway in mammals, such as the prototypical cytoprotective enzyme NAD(P)H dehydrogenase 1 (NQO1). **Methods:** Eighteen healthy human volunteers (9 males, 9 females, aged 18–69. were randomized to receive daily glucoraphanin (GR), which is converted to SF upon ingestion (450 mg; 1 mmol), CUR (1000 mg; 2.7 mmol), or both (450 mg GR + 1000 mg CUR), as oral supplements. After 8 days of a diet low in both compounds, blood and urine were collected for compliance and biomarker measurements. Randomized spots on the buttock’s skin were exposed to 2 x M.E.D. of UVB, and punch biopsies were obtained 1 and 3 days later for biomarker and histological measurement. Erythema was measured with a chromameter daily for 3 consecutive days following UVB. The process was repeated after receiving oral supplements, both with and without UVB exposure. **Results:** Compared to baseline, each treatment (*n* = 6 for each) induced NQO1 mRNA levels in skin biopsies: 3.1-fold with GR, 3.3-fold with CUR, and 3.6-fold with the combination of GR and CUR. Across all treatments (*n* = 18), expression of the pro-inflammatory cytokines IL-1β and TNF-α were reduced, as were IL-6, IL-17, STING, and CYR61, though less robustly. Modulation of these biomarkers persisted, but was less pronounced, in biopsies taken following UV exposure. The presence of SF and its metabolites in the skin post-treatment was confirmed by examining 6 of 12 subjects who ingested GR. Supplement effects on erythema following UV exposure were not significant, and no significant changes were measured in the same biomarkers in blood cells (PBMC), or by counting dyskeratotic keratinocytes. Supplements were well tolerated and compliance was excellent. **Conclusions:** Oral GR and CUR are well tolerated and have for the first time been shown to result in increased expression of cytoprotective genes and reduced expression of inflammatory cytokine genes in human skin in vivo. This mechanism-based clinical study suggests that an antioxidant, anti-inflammatory, and cytoprotective benefit from these oral supplements is delivered to the skin in humans.

## 1. Introduction

Oxidative stress plays a pivotal role in skin aging and carcinogenesis, whether incited by extrinsic factors such as ultraviolet (UV) radiation, or intrinsically via cellular oxidative metabolism [1]. Both antioxidant and anti-inflammatory agents have long been invoked in the search for ways in which to protect skin from both aging and carcinogenesis. Plants (both edible and not) produce millions of compounds in low abundance (unlike fats, carbohydrates, proteins, fiber, and vitamins) that are collectively known as phytochemicals or phytonutrients. Some of these phytochemicals have potent antioxidant and/or anti-inflammatory potential as has been widely demonstrated in the literature [2], in particular, glucoraphanin (GR) from broccoli, and curcumin (CUR) from turmeric. Phytochemicals are an integral but much-overlooked part of our fresh food supply. Many have dramatic potential to enhance human health [3,4], and both GR and CUR can now also be delivered in concentrated form as supplements.

Orally delivered sulforaphane (SF), supplied either directly, or as its biogenic precursor GR has been evaluated for its pharmacokinetics, bioavailability, and potency in cytoprotection. It does so by upregulating the Keap1 (Kelch-like ECH-associated protein 1)-Nrf2 (nuclear factor-erythroid 2 p45-related factor 2) nuclear transcription pathway, inhibiting the pro-inflammatory NF-κB cascade, up-regulating heat-shock proteins, inhibiting histone de-acetylation, and reducing advanced glycation end-products (AGEs), to name only some of the major recognized modes of action of SF [5,6,7,8,9,10,11]. SF has also been evaluated for its potential to ameliorate or prevent symptoms of autism, air pollution injury, chronic obstructive pulmonary disease (COPD), asthma, and chemically induced liver toxicity in human volunteers [12,13,14,15,16,17,18,19].

In dermatologic studies, SF has been shown to provide robust protection against the erythema induced by UVB or simulated solar irradiation when topically applied to the skin of healthy human volunteers [20,21,22]. In animal models, these observations have been extended to include protection against skin tumorigenesis [23,24,25]. The only study in which SF (given orally as its GR precursor) reduced skin carcinogenesis, was performed in a mouse model [25]. There are only two studies we are aware of in which oral SF was evaluated for its effect on a dermal condition in humans. The first reported positive effects on melanoma (atypical nevi) following 50, 100, or 200 micromoles of SF in broccoli sprout extract, daily, for 28 days [26]. The second reported Keap1-Nrf2 mediated amelioration of skin aging [27]. To our knowledge, nobody has evaluated the effect of oral SF on erythema in human beings.

CUR is another potent cytoprotective dietary phytochemical that has many overlapping, and some non-similar modes of action as GR /SF. CUR has been a mainstay of Indian traditional (Ayurvedic) medicine for centuries due to its antibacterial, antifungal, and anti-inflammatory activities. Its antineoplastic properties are manifold and powerful, and its safety is well established at doses of up to about 12 g per person per day. CUR has been shown to be highly protective against inflammation in a wide range of experimental systems by reducing COX, NF-κB, lipoxygenase, LTB4, LTC4, PGE2, TNF-α, and IL-6 [28,29]. We and others have conducted animal and clinical studies with CUR and its analogs [30,31,32].

The dermal application of CUR has historically been problematic, and its bioavailability is also very poor when delivered orally due to its very hydrophobic nature. That aside, CUR has been shown to be effective when given topically for the treatment of mastitis in breastfeeding women [33], and orally for the amelioration of radiation dermatitis in breast cancer patients [34]. We have evaluated the protective effects of topical CUR treatment in the same tumor-prone SKH1 mouse model previously referenced [25], and it protects from UV-induced carcinogenesis in mice (Fahey, unpublished results).

In this study, we investigate whether orally administered phytochemicals (or their metabolites) can be detected in the skin of subjects after dosing. This is assessed using skin punch biopsies collected from UVB-naïve sites before and after phytochemical dosing. We also evaluate, regardless of the detection of the compounds themselves in the skin, whether GR, CUR, or their combination modulate the expression of genes associated with cytoprotection (detoxication and antioxidant) and/or inflammation in the skin. This analysis is conducted using RNA extracted from a portion of the same biopsies. We assess for the presence of the SF metabolites in the urine of study subjects as a marker of compliance. Additionally, we evaluate the same biomarkers of cytoprotection and inflammation using peripheral blood mononuclear cells (PBMCs). Finally, we examine whether these changes caused by GR, CUR, or their combination confer protection against UVB-induced skin damage (erythema), by clinical assessment, measurement of dyskeratotic keratinocytes in biopsies, direct digital photography, and chromametry.

## 2. Materials and Methods

### 2.1. Study Design

This was a pilot, single-center, randomized controlled trial to study the effect of two phytochemicals (GR and CUR) on UVB-induced cutaneous changes. The study consisted of a non-intervention phase followed by an intervention phase. It was performed in accordance with the Declaration of Helsinki, approved by the institutional review board at Johns Hopkins University, and all participants provided written informed consent. Exemption to a requirement for a full IND was issued by the FDA (PIND 134812), and the study is registered at clinicalTrials.gov, accessed on 1 May 2025 (ID NCT03289832). Study procedures were performed at the Johns Hopkins Hospital, Baltimore, MD, USA.

### 2.2. Patient Population

Eighteen healthy adult volunteers (9 males and 9 females) between the ages of 18 and 69 years, with skin types I and II, were recruited from the dermatology clinics at the Johns Hopkins Health System and the surrounding community between September 2017 and September 2019. Subjects were asked to refrain from consuming cruciferous vegetables and condiments that might contain glucosinolates or isothiocyanates or CUR and to avoid exposure of the buttocks to sunlight for 3 days before and during each phase of the study. Exclusion criteria consisted of medications that cause photosensitivity or skin flushing, use of anticoagulants/antiplatelet therapies, allergies to anesthetic agents, use of systemic retinoids or steroids (excluding female contraceptives and levothyroxin), use of topical retinoids or steroids at study sites, and use of antibiotics. Also excluded were current students of the principal investigator, subjects with procedures performed at the study sites, smokers/tobacco users, and inability to comply with the study dietary and sun exposure restrictions. A CONSORT diagram is included as Supplementary Figure S1.

### 2.3. Randomization

**Figure 1:**
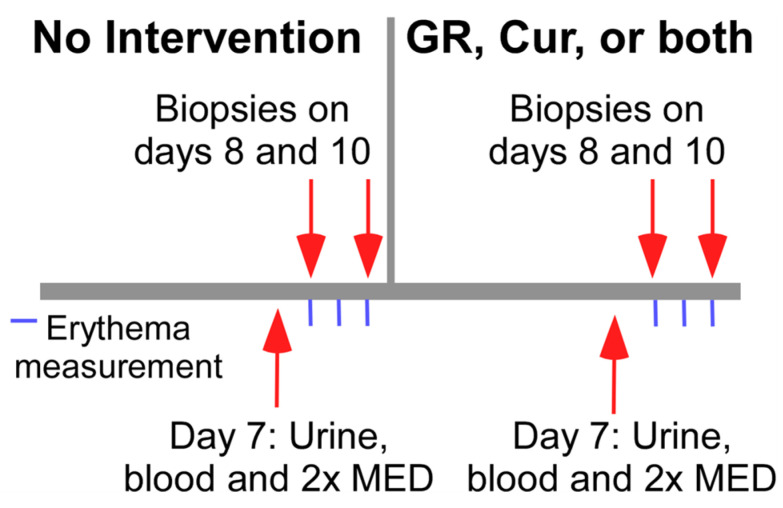
Study design. Each patient went through a non-intervention followed by an intervention phase, 10 days each. Urine and blood were obtained, and irradiation was performed on day 7 of each phase. Erythema was assessed at days 8, 9, and 10 of each phase, relative to baseline skin color at day 7. Punch biopsies were obtained on days 8 and 10.

Subjects were randomized using a computer-generated randomization scheme into 3 groups of 6 subjects each, to receive daily: (a) Crucera-SGS^®^ with TrueBroc^®^ broccoli seed extract (Brassica Protection Products, LLC, Baltimore, MD, USA) as a source of GR that is converted to SF; 9 capsules (450 mg or 1.03 mmol GR) per day; (b) Meriva 500-SF^®^ as a source of CUR; 2 capsules (1000 mg or 2.72 mmol total curcuminoids) per day; or (c) Crucera-SGS^®^ (9 capsules) plus Meriva 500-SF^®^ (2 capsules). Each intervention group served as its own control as the same subjects went through a non-intervention followed by an intervention phase (Figure 1).

### 2.4. Dose Procurement and Validation

Single lot shipments of each of the study interventions (Crucera-SGS^®^ and Meriva 500-SF^®^) were donated by Thorne Research, Inc. (Summerville, SC, USA) along with their respective certificates of analysis (COA). Supplements arrived in Baltimore in unopened boxes and jars of commercially available supplements. Analyses were conducted by HPLC and spectrophotometry on five separate extracts made from individual gelcaps (of each) in appropriate solvents, after the removal of non-dissolved particles. Contents of GR and CUR were determined to be as certified on their respective COAs.

### 2.5. UV Treatment

Each subject underwent both non-intervention and intervention phases, ten days per each. On the seventh day of both phases, subjects fasted overnight and provided urine and blood samples before receiving a single treatment of UV-radiation to the left or right buttock, corresponding to two times the minimum erythematous dose (M.E.D.), using the Lumera UVB light phototherapy device (Daavlin, Bryan, OH, USA) (emission spectrum 290–320 nm).

### 2.6. Clinical Assessment

In both phases, following 2 x M.E.D. treatment on day 7, skin color was measured at the irradiation sites daily for 3 consecutive days (days 8–10) using a hand-held chromameter (Konica Minolta CM-2600d, Osaka, Japan), which uses the L*a*b* color system determined by the Commission International de I’ Eclairage. L* gives information about the black-white axis, ranging from black = 0 to white = +100, and a* represents the balance between red (+100) and green (−100) [35]. Digital photographs were also obtained.

#### 2.6.1. Specimen Collection

- Blood and Urine. Following the 7th dose of either GR, CUR, or both, the subjects collected and supplied their entire production of urine excreted during the preceding 24 h for evaluation of SF metabolites. They were instructed to collect for the first 8 h, and the subsequent 16 h, in separate containers. Blood was drawn from the participants on day 7 of both the non-intervention and intervention phases and processed for peripheral blood mononuclear cells (PBMC) isolation [12]. Briefly, 8 mL of whole blood was drawn from the participants into Vacutainer CPT tubes (Becton, Dickinson and Company, Franklin Lakes, NJ, USA) at room temperature, centrifuged, and PBMCs were washed twice with phosphate-buffered saline. PBMC pellets were stored at −80 °C for RNA extraction.
- Biopsies. Two 6-mm skin punch biopsies were taken approximately 24 h after UV irradiation (one from a UV-irradiated area, and the other one from a vicinal non-irradiated area), on day 8 of both phases. Individual biopsies were immediately placed in cryotubes, snap-frozen in liquid nitrogen, and stored at −80 °C. Immediately prior to RNA extraction, each frozen biopsy was cut into halves. One half was reserved for dithiocarbamates (DTC) measurement, and the other for RNA extraction. Additional biopsies were taken after 3 days of UV irradiation (Day 10 of each phase) and embedded for H&E staining and subsequent measurement of dyskeratotic keratinocytes.

#### 2.6.2. Laboratory Studies

- Total RNA Extraction and Quantitative Real-Time PCR. Frozen skin biopsies were pulverized in liquid nitrogen and homogenized in 0.6 mL of Buffer RLT (from RNeasy mini kit, QIAGEN, Valencia, CA, USA), and then were transferred to QIAshredder (QIAGEN, Valencia, CA, USA). After centrifugation at 14,000 rpm for 2 min, the homogenized lysates were used for RNA extraction using an RNeasy mini kit. Total cellular RNA was also extracted from frozen PBMC using QIAshredder and RNeasy mini kit [12]. Synthesis of cDNA and quantitative real-time PCR was performed as described [12]. Primer sequences for gene amplification are provided in Supplementary Table S1.
- Biomarkers. To evaluate the effect of GR and CUR on cytoprotective mechanisms, we measured mRNA levels of markers of the Keap1/Nrf2-linked cytoprotective phase 2 response, the inflammatory response, and matrix metalloproteinase activity, following 7 days of oral supplement intake. Measurements were made both in blood and in skin samples harvested from the same subjects. All comparisons were made to baseline samples taken from the same individuals just prior to ingestion of the first supplement doses. Gene targets and their functions are provided in Supplementary Table S2.
- Preparation of Biopsies for DTC Measurement. Frozen biopsies were pulverized into powder under liquid N_2_. The powder was weighed and about 30 mg was resuspended in ice-cold buffer (100 mM potassium phosphate, pH 7.4; 100 mM KCl; 0.1 mM EDTA), homogenized in an ice bath, and subjected to centrifugation at 4 °C (15,000× *g* for 10 min) [23].
- Cyclocondensation. This assay measures DTC, which is the sum of SF and its conjugation products, and it has a limit of detection of 5 pmol. Cyclocondensation was performed on both urine and pulverized biopsied skin according to published methods [36] and was used to confirm compliance and assess bioavailability with treatment assignments.
  1. Urine: Total DTC excretion in each urine sample (three per subject) and urine creatinine concentrations were determined, as an indicator of compliance.
  2. Blood: Measurement (HPLC) of CUR and its major conjugates, curcumin glucuronide, and curcumin sulfate, in plasma samples, was attempted, but as expected did not yield useful data.
  3. Skin: An a priori decision was made not to process biopsies from the “curcumin, only” treatment arm. However, due to a −80 °C freezer loss associated with lab closure, we were able only to process 6 of the 12 samples in the treatment arms that had received GR (the GR and GR + CUR arms), as 6 of the tissue samples remaining after biopsies had been split for RNA extraction were lost.
- Measurement of Dyskeratotic Keratinocytes. Embedded 6 mm biopsies taken on Day 10 were sectioned and standard hematoxylin and eosin (H&E) were performed. Epidermal thickness (taken from the top of granular layer to the basal layer) was measured using an Olympus BX41 microscope and a number of dyskeratotic keratinocytes (DK) was scored per epidermal area (EPI) to create an index DK/EPI.

### 2.7. Data Analysis and Statistics

#### 2.7.1. Clinical Assessment

Erythema and DK/EPI scores were compared between the non-intervention and intervention phases for each intervention group using a two-tailed student *t*-test with statistical significance set at *p* < 0.05.

#### 2.7.2. Biomarker Evaluation

Gene expression was calculated using the comparative 2^−ΔΔCT^ method [12,37], and differences between pre-dose and post-dose expression levels of each tested marker were evaluated by two-tailed paired *t*-tests. Differences in grouped markers from baseline were analyzed by a one-sample mean comparison test. Statistical significance was set at *p* < 0.05.

## 3. Results

### 3.1. Study Subjects and Compliance

This study included 18 subjects: 9 females and 9 males (ages 18 to 69 years, avg (SD) 38.9 (16.7) years). The average age was 47 (17.1) years in the GR group, 33.2 (13.6) years in the CUR group, and 36.5 (18.7) years in the combination group (Figure 1 and Table 1). Females represented 16.7% in the GR group, and 66.7% in the other 2 groups (Table 1).

**Figure 2:**
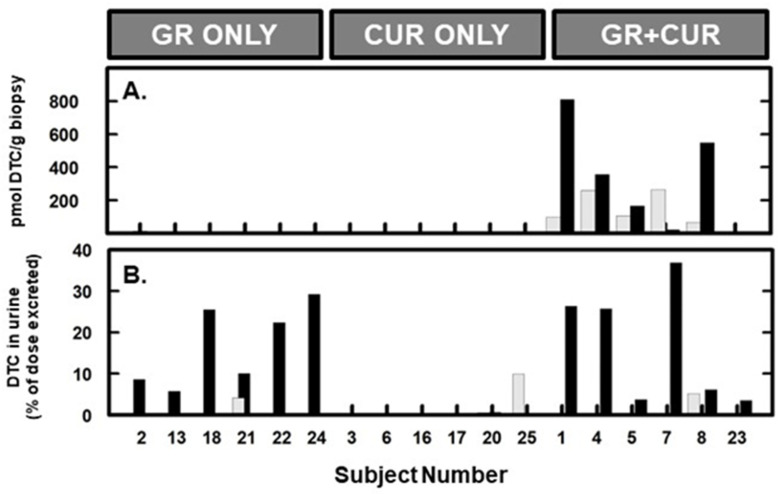
DTC measured pre- and post-dose: in skin (**A**) and urine (**B**). Shaded bars represent pre-dose baseline measures, and solid bars represent samples taken post-dose as described in the text. Treatment assignments are provided at the top of the figure and subject numbers are those that were assigned at enrollment. (**A**) Pre- and post-treatment skin biopsies could only be assessed on 6 subjects; Subject 2 received only GR (but DTC was below limits of detection both pre- and post-dose), and subjects 1, 4, 5, 7 & 8 received GR + CUR. (**B**) Pre- and post-dose urines were collected and measured for all subjects; “Percent of GR dose excreted” is utilized (left x-axis) as a means to compare all treatment arms, however the “curcumin, only” arm did not receive any GR. The positive result in the baseline urine of subject #25 in the “curcumin, only” arm most likely represents a dietary infraction.

Compliance was excellent, and 100% of the subjects completed the study. Treatments were well tolerated: one subject in the GR group experienced nausea starting on the third day of the intervention phase, and loose stools at day 7, but no pain, vomiting, or other discomforts. They reported taking Imodium^®^ with prompt resolution of symptoms. The subject was given the choice to stop taking treatment (GR) but chose to continue and complete the study. Compliance was also validated by measurement of urinary DTC, which are indicators of consumption of SF and other isothiocyanates. These are distinctive components of cruciferous vegetables, which were prohibited by the study protocol and lists of these vegetables and foods that likely contained them were given to each subject at the time of consenting. DTC were not present or at extremely low levels pre-intervention, and reached varying levels in all subjects who received GR either alone or in combination with CUR (Figure 2). Mean urinary excretion (conversion or bioavailability) was 17% of the GR dose (*n* = 12), a level highly consistent with other reports in the literature [38,39,40]. On average excretion was identical whether GR was delivered by itself or with CUR.

### 3.2. Outcomes

#### 3.2.1. UV-Induced Erythema

**Figure 3:**
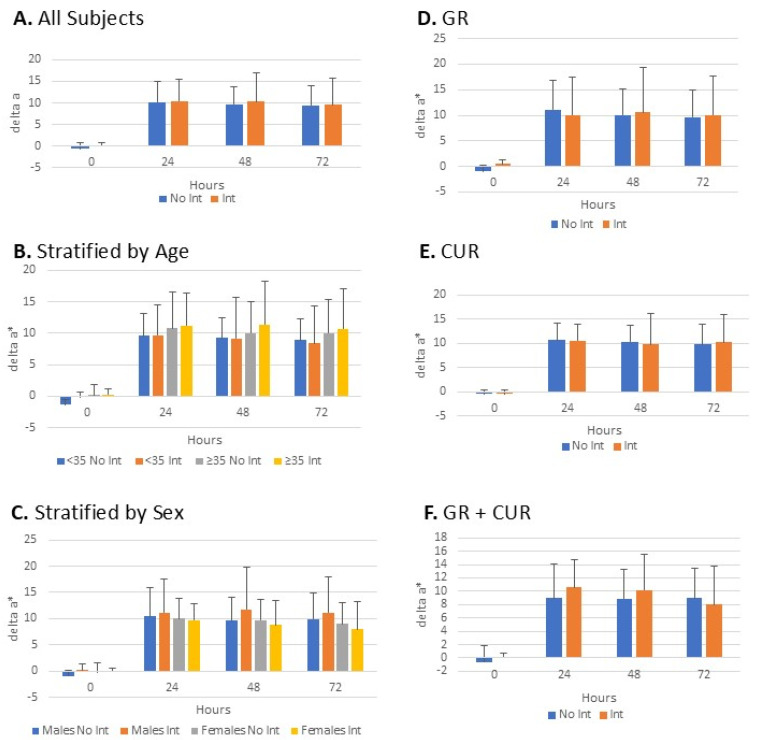
Skin erythema. Erythema was measured at 24, 48, and 72 h after UVB irradiation in each of the non-intervention and the intervention phases (**A**). Subgroup analyses were then performed by stratifying patients based on age (**B**), sex (**C**), and type of intervention: GR (**D**); CUR (**E**); GR + CUR (**F**). Only Δa*, measures are shown.

A significant increase in erythema (a*) was observed at 24 h after UV irradiation, and this persisted at 48 and 72 h (Figure 3A). There was no difference in erythema induction or duration between the non-intervention phase and intervention phase of all treatments combined (Figure 3A). Stratification of subjects by age (above and below the median of 35 years), showed the same degree of erythema induction and duration regardless of treatment or age (Figure 3B). Erythema was also comparable between the intervention and control phases in both males and females (Figure 3C). Subjects were then stratified into 3 groups based on intervention type (GR, CUR, or both). Similarly, the magnitude of erythema and its duration were comparable between both study phases in all 3 groups (Figure 3D–F). L* values showed similar trends as the a* values.

**Figure 4:**
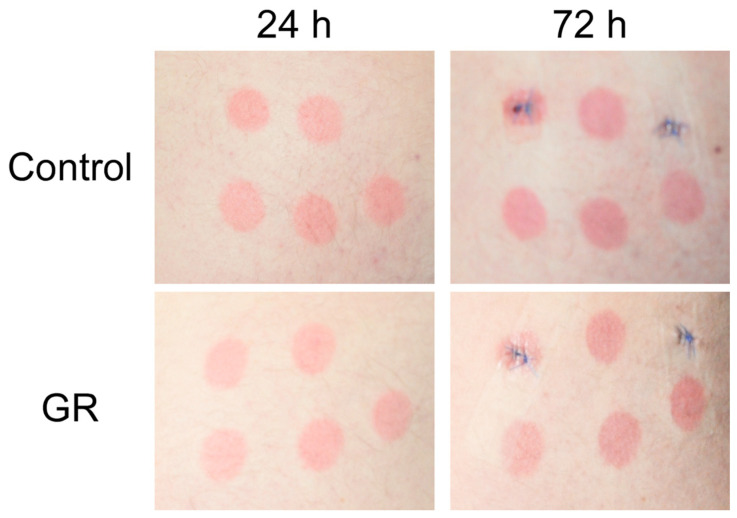
UVB-induced erythema. Sample images of erythema in irradiated skin at 24 and 72 h after UVB irradiation. This subject received GR in the intervention phase.

Representative images of erythema at 24 and 72 h are displayed in Figure 4. In an effort to determine whether differences between subjects existed that could not be detected by chromametry, we measured dyskeratotic keratinocytes to assess for acute photodamage changes in representative epidermal sectors from each biopsy at 72 h after UV-irradiation (designated as Day 10, in Figure 1). These products of UV-mediated cell damage (“sunburn cells”) give an alternative, microscopic view of skin damage and its amelioration. On average across all 3 treatments (GR, CUR, and GR + CUR) there was a 24% reduction in the increase in the number of newly formed dyskeratotic cells caused by UV treatment, but these differences were not significant (*p* = 0.09 by *t*-test) (Supplementary Figure S2).

#### 3.2.2. Biomarker Modulation

After only 7 days of supplement intervention, both GR and CUR induce the cytoprotective response and reduce pro-inflammatory cytokines in the skin, but not in blood cells. A description of their function and synonyms, abbreviations, common names, and general categories is provided in Supplementary Table S2.

Blood. Supplement consumption produced no significant systemic changes in 12 biomarkers measured in isolated PBMC after 7 days of supplementation. (Supplementary Figure S3).

**Figure 5:**
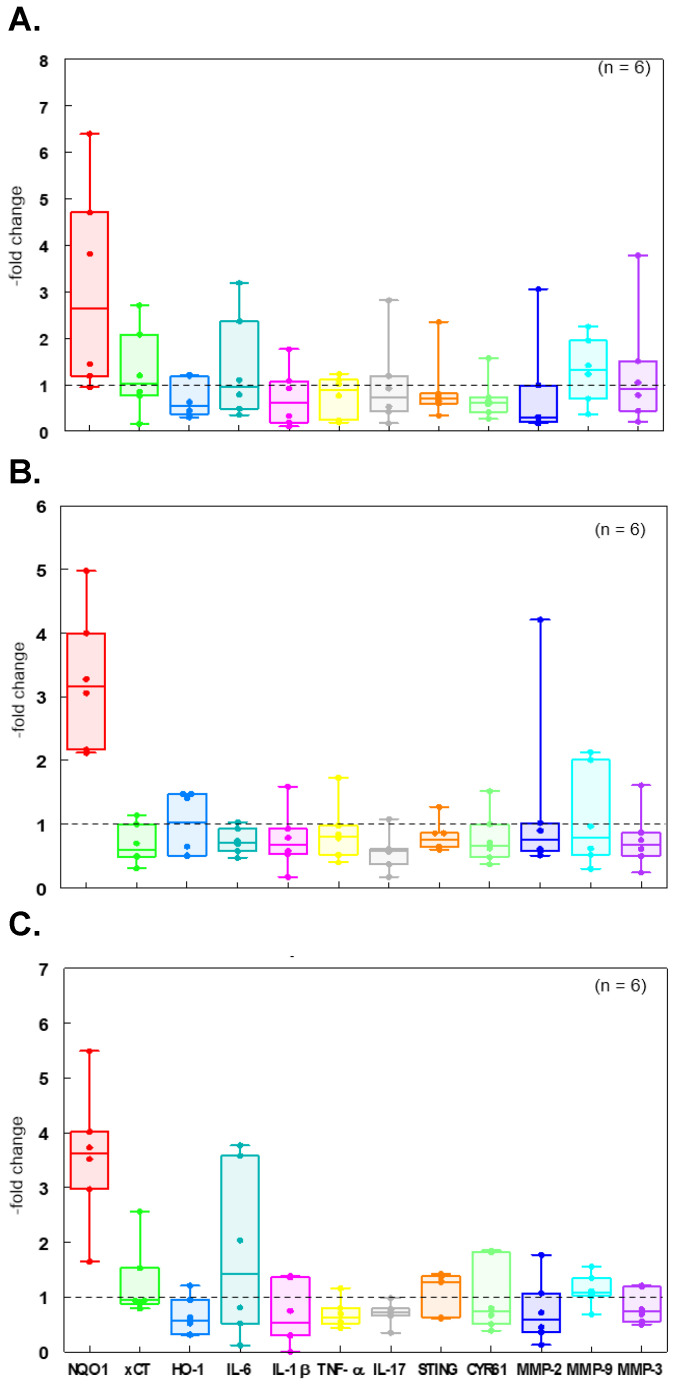
Relative gene expression in skin-punch biopsies from non-UVB irradiated areas, taken a week after daily oral supplementation. Each data point (*n* = 6 subjects per supplement treatment) represents a comparison (“fold-change”) to expression from a control biopsy taken from the same subject on the 8th day of the non-intervention phase. Boxes envelop the 25th to 75th percentile of the data, whiskers are at the 5th and 95th percentile, and horizontal lines within boxes denote medians. (**A**) GR, (**B**) CUR, (**C**) GR + CUR.

Skin. In contrast to the lack of effect in blood cells, there was a striking and significant increase in mRNA copy number for NQO1, in skin biopsies taken after a week of daily oral supplement consumption, when compared to baseline biopsies taken prior to supplement consumption. The increase was 3.1-fold with GR, 3.3-fold with CUR, and 3.6-fold with the combination of GR and CUR (*p* < 0.05) (see Figure 5A–C, respectively).

**Figure 6:**
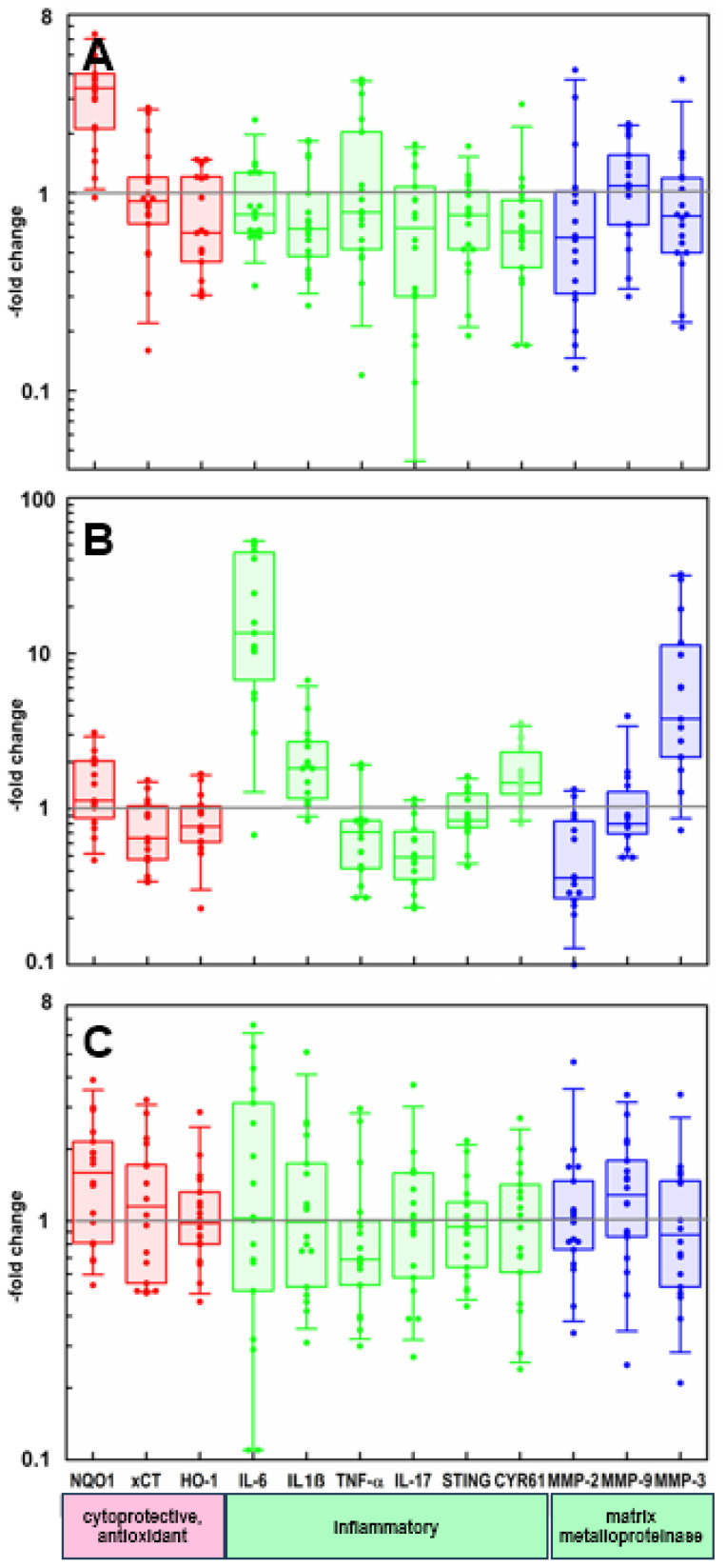
Relative gene expression in skin-punch biopsies. Each data point (*n* = 18 subjects) represents a comparison (“fold-change”) to expression from a control biopsy taken from the same subject. Boxes envelop the 25th to 75th percentile of the data, whiskers are at the 5th and 95th percentile, and horizontal lines within boxes denote medians. Corresponding means and statistics are provided in Table 2. Results are grouped and color-coded according to predominant gene function. (**A**) Following one week of daily oral supplementation. Biopsies were taken from a non-UV-irradiated area. (**B**) Biopsies taken 1 day following a single UVB irradiation of subjects who had not taken any supplements (and compared to non-irradiated skin from the same individual). (**C**) Biopsies taken 1 day following a single UVB irradiation of subjects who had taken supplements (and compared to irradiated skin from the same individual).

When changes in gene expression were examined across all 18 subjects (all three treatment arms), the increase in NQO1 (3.3-fold) expression was highly significant by one-sample mean comparison test (t = 6.4; *p* < 0.0001) (Figure 6A and Table 2). Furthermore, expression of HO-1, IL-1β, and TNF-α were reduced by 22% (*p* = 0.024), 28% (*p* < 0.024), and 23% (*p* < 0.013) (Figure 6A and Table 2).

Compared to non-irradiated control skin, a single treatment with UVB (2 x M.E.D., 24 h later) enhanced expression of the proinflammatory cytokines IL-6, IL-1β, and CYR61, as well as MMP-3, and reduced expression of IL-17 and MMP-2 (Figure 6B).

In UVB-treated skin, a comparison of the effect with and without one week of daily oral supplement intervention showed no significant modulation of any of the biomarkers examined (Figure 6C; Supplementary Figure S4). Although values ranged widely between individuals, when grouped by functionality (cytoprotective and antioxidant (1), inflammatory (2), and matrix metalloprotein (3)), all 3 functional groups were significantly changed upon UVB exposure with supplement consumption (Table 2).

We could not measure blood, urine, or skin biopsy levels of CUR post-dose. CUR is rapidly metabolized and our methodologies were not sensitive enough to detect these metabolites. However, we were able to assess GR metabolites in the skin of 6 subjects (Figure 2A). Levels rose dramatically in the skin of 4 of the 6 subjects evaluated after dosing.

## 4. Discussion

In this study, we investigated the bioavailability of oral GR (SF precursor) and CUR in the skin and evaluated their ability to protect from UV-induced oxidative stress, erythema, and mutagenesis. Doses of each compound were chosen to be at the high end (6.3 and 14.2 mg/kg, respectively), of what has typically been safely administered in previous clinical studies, in order that we might be as likely as possible to see effects in the skin. We found that both supplements were well-tolerated and resulted in increased expression of cytoprotective genes and reduced expression of inflammatory cytokine genes in human skin in vivo. This is exciting as it is the first time such an effect has been shown in vivo, though these effects have been very widely reported in keratinocytes for both compounds, in both in vitro and preclinical systems.

Bioavailability of both SF and GR when delivered ***orally*** has been the subject of much study however these studies evaluated the presence of the compound and/or its metabolites in the blood, urine, or stool, but ***not*** in the skin [38,39,40,41,42], and only 2 of them examined biomarkers in the skin [26,27]. Most studies of SF bioavailability have to date been performed on healthy volunteers not taking drugs or supplements. In these studies, very high bioavailability of SF was reported, whereas substantially lower bioavailability was reported when GR was delivered [8,9,38,39,40,42]. However, since GR is converted to SF in the body, increasing the GR dose can deliver comparable amounts of SF as measured in blood and urine. The oral bioavailability of CUR is very low but has been greatly enhanced in a widely sold nutritional supplement (which has been used in this study). A variety of clinical studies have demonstrated both enhanced bioavailability and equivalent efficacy of this phospholipid-formulated product, to the parent compound [43,44,45,46,47,48].

CUR, as well as SF and its metabolites, are typically taken up in somatic tissues where they exert their protective effects [20,49,50,51]. The blood residence time of both phytochemicals administered in this study is very short. Despite that, our demonstration of increased levels of the GR metabolites SF and its glutathione-derived conjugates (measured collectively as DTC) in skin tissue was remarkable, and to our knowledge, it is the first time that SF and its metabolites have been demonstrated in human skin following oral consumption of GR. It is further encouraging that this occurred after only a week of dosing. It should be noted that whereas there was a single non-detect, as well as a decline in DTC in one subject, 4 of the 6 subjects whose biopsy tissue we tested showed substantial increases after 7 days of supplement ingestion. The fact that they had measurable DTC prior to supplementation is neither surprising nor concerning, as this was likely a result of prior accumulation from cruciferous vegetables in the diet of those individuals prior to the study (they were forbidden from eating them during the study).

The cytoprotective effects of SF from broccoli, broccoli sprouts, broccoli seeds, and CUR from turmeric have been well established [6,8,10,28]. Among the major mechanisms implicated is the induction of the cytoprotective Keap1-Nrf2-ARE nuclear transcription pathway [9,11,30,52]. Nrf2 is a redox-sensitive transcription factor that is a key player in the body’s response to cellular stress. Under normal-stressed conditions, Keap1 anchors the Nrf2 transcription factor within the cytoplasm, targeting it for ubiquitination and degradation. In response to a variety of cellular stressors, Keap1 undergoes a conformational change that alters its ability to anchor Nrf2, allowing it to pass into the nucleus where it binds to the antioxidant response element (ARE). ARE is located in the promoter region of a collection of downstream genes encoding cytoprotective and antioxidant phase 2 proteins (enzymes) such as NQO1. Thus, through the induction of this and other signaling pathways, both SF and CUR can up-regulate a large number of cytoprotective and antioxidant enzymes.

In addition to KEAP1-Nrf2-associated biomarkers, we chose the biomarkers to be evaluated based on knowledge of metabolic pathways that are either activated or suppressed by UV exposure [53]. In particular, we focused on pathways downstream of NF-κB, which is induced by UV exposure and in turn upregulates pro-aging cell surface cytokine expression, e.g., IL-1, and TNF-α [53]. The choice of IL-17 was conditioned by abundant evidence of its involvement in a wide range of inflammatory, autoimmune, and oncological skin diseases [54]. The intimate involvement of transcription factor activator protein 1 (AP-1) in collagen synthesis and breakdown prompted our inclusion of cysteine-rich 61 (CYR61) protein, which is an inducer of AP-1 and a regulator of UV-triggered collagen synthesis [53]. As a result of evidence from AP-1 and MAP kinase-mediated responses to oxidative stress, we targeted the matrix metalloproteinases (MMPs) MMP-2 and MMP-9 (both gelatinases and both of which are connective tissue degrading enzymes), as well as MMP-3 (a collagenase) along with IL-1β [55,56,57,58]. Another biomarker we targeted was the stimulator of interferon genes (STING) which has recently been implicated in radiation-induced tissue damage associated with inflammasome-knockout and hyperactivation of a cyclic GMP-AMP synthase-STING innate immune response [59]. Finally, we included IL-6 in our query of biomarkers because of demonstrations of its dramatic and dose-dependent elevation in solar-simulated radiation experiments [60].

A significant increase in erythema was observed at 24 h after UV irradiation, and this persisted at 48 and 72 h. There was no difference in either the induction or duration of erythema between the non-intervention and intervention phases of all supplement treatments combined even when the data was stratified as discussed previously.

It is possible that the duration of supplement treatment was not sufficient to translate into appreciable clinical alterations but adequate to induce selected molecular changes in the targeted tissue. One of the key changes we found was in the key detoxification and indirect antioxidant enzyme NQO1, a well-documented target of both SF and CUR via the KEAP1-Nrf2 pathway, following seven days of treatment. This was elevated following the intervention phase for all three arms.

We were also able to show that only one day after UVB irradiation, there were changes in the gene transcription levels of some of the known biomarkers of inflammation, and of tissue remodeling and physical adaptation (MMPs). Changes in these groups of biomarkers can be a result of developmental, environmental, or disease-related cues.

Compared to non-irradiated control skin, a single treatment with UVB led to enhanced expression of IL-6. This inflammatory cytokine is known to be upregulated by NF-кB, which is induced by reactive oxygen species following ultraviolet light exposure. IL-6 in conjunction with other cytokines also induced by this mechanism further activates and sustains the NF-кB pathway. We found a reduction in IL-6 following intervention in our study demonstrating that these oral agents may offer protection against the inflammatory cascade that follows after sun exposure.

The formation of reactive oxygen species following UV exposure also activates transcription factor activator protein a (AP-1). AP-1 increases the production of matrix metalloproteinases (MMPs) such as MMP-3, which was found to be elevated following UV exposure in our study. MMPs degrade types I and III collagen in the dermis thus accounting for the breakdown of the dermal matrix leading to the features of photoaging. We found that MMP3 decreased following the use of the supplements in this study, highlighting the potential role SF and CUR can play in warding off the breakdown of the dermal matrix with solar irradiation.

It is important to note that there is data now pointing to possible gaps in the understanding of the role MMPs play in photoaging. It is a long-held understanding that UV-induced AP-1 temporarily upregulates MMP-1, -3, and -9. In sun-exposed skin, these MMPs are present at low levels, do not always correlate with wrinkles, and thus may not tell the entire photoaging story. Recent data show that MMP-2, which breaks down type IV collagen (makes up the skin basement membrane zone), may play a role in this process. It is found in high levels in the skin and increases with age in sun-exposed skin, where it correlates well with aryl hydrocarbon receptor (AhR), a transcription factor activated by UV-generated metabolites, and inhibits nuclear excision repair function. The resulting DNA damage leads to a cascade of events that ultimately leads to photodamage [61,62].

We did not observe significant changes in MMP-2 in our study, and this may be due to the design of the study as MMP-2 changes post ultraviolet light exposure may require more time to manifest. We did assess for dyskeratotic keratinocytes as a surrogate marker of acute sun-related damage but did not see significant changes. There are likely multiple pathways at play in UV-induced skin damage that require further elucidation.

Our inability to resolve any differences in blood (PBMC) biomarkers was also not surprising, based on the large body of literature showing an enhanced cytoprotective response in target tissues rather than in the circulatory system. Furthermore, the study was not powered to assess changes in blood levels of these markers. However, in a recent pilot study for a clinical trial of SF in children with autism spectrum disorder, the expression of cytoprotective markers (NQO1, AKR1C1, HO-1, HSP70, and HSP27) increased and the expression of some pro-inflammatory markers (IL-6, IL-1β, COX-2, and TNF-α) decreased in PBMC after a longer SF treatment time (2 weeks). Although the change of most individual markers did not reach statistical significance, the functionally grouped markers showed highly significant differences from the baseline [12].

The ability of oral nutritional supplements to reach the skin and to selectively modify the expression of important biomarkers has dramatic implications for protection against aging, cancer, and other conditions. Though following a single UVB irradiation, we did not see overall supplement-induced short-term modulation of the dozen biomarkers we had targeted: (a) The UVB dose was rather high (2 x M.E.D.) and it led to rapid and unambiguous development of erythema. Responses to a lesser dose would be interesting to evaluate, as even sub-M.E.D. UV irradiation has been shown by others to induce rapid upregulation of certain inflammatory cytokines. (b) A one-week course of daily oral supplements at a reasonably high level was used for the supplementation phase of the study, but a longer supplementation could well have resulted in greater targeting of the skin (e.g., accumulation) and in a more robust effect both without- and with- added UVB insults. A larger sample size would of course also have resulted in a more homogeneous post-randomization distribution by sex and age. It could also have better illustrated the effects of these supplements following UV exposure since we previously showed that with an identical dose of both UVB and SF applied topically, there are clear differences in protective response among individuals [20].

## 5. Conclusions

To our knowledge, this study is the first time that SF and its metabolites have been demonstrated in human skin following oral consumption of GR. GR and CUR are well-tolerated when taken orally, and GR metabolites in the skin increase significantly following only one week of consumption. Furthermore, both oral supplements resulted in increased expression of cytoprotective genes and reduced expression of inflammatory cytokine genes in human skin in vivo. The data highlight the vital role that oral photoprotective agents can play in combating the photocarcinogenesis and photoaging effects of ultraviolet irradiation, perhaps as an alternative to, and certainly as an adjunct to sunscreens and topical antioxidants.

## Figures and Tables

**Table 1:** Baseline characteristics and adverse events in each treatment group.

	GR (*N* = 6)	CUR (*N* = 6)	GR + CUR (*N* = 6)
Age, years AVG (SD)	47 (17.1)	33.2 (13.6)	36.5 (18.7)
Sex, females, *N* (%)	1 (16.7)	4 (66.7)	4 (66.7)
Attrition, *N* (%)	0	0	0
Adverse events, *N* (%)	1 (16.7) *	0	0

* Subject experienced nausea on day 3 and loose stools on day 7 of the intervention phase, all symptoms resolved by day 10 with symptomatic management (Imodium^®^). Subject given the option to discontinue treatment but chose to continue and completed the study.

**Table 2:** Skin punch biopsy expression of biomarker genes, and functional groups of genes, relative to their baseline expression. Subjects were taking supplements (A & C) or not (B), and biopsied skin was exposed to UV (B & C) or not (A).

Gene or Functional Grouping	*N*	Mean	StdErr	t	Pr (T < t)	Pr (T > t)
No UV + Supplements						
NQO1	18	3.361	0.360	6.404	1.000	0.000
xCT	18	1.086	0.168	0.512	0.692	0.308
HO-1	18	0.784	0.102	−2.123	0.024	0.976
IL-6	18	1.309	0.273	1.132	0.863	0.137
IL-1β	18	0.728	0.127	−2.138	0.024	0.976
TNF-α	18	0.777	0.091	−2.445	0.013	0.987
IL-17	18	0.759	0.139	−1.737	0.050	0.950
STING	18	0.943	0.112	−0.509	0.309	0.691
CYR61	18	0.831	0.120	−1.416	0.087	0.913
MMP2	18	0.964	0.253	−0.143	0.444	0.556
MMP9	18	1.182	0.144	1.266	0.889	0.111
MMP3	18	0.960	0.190	−0.211	0.418	0.582
Cytoprotective/antioxidant	54	1.725	0.205	3.545	1.000	0.000
Inflammatory	108	0.891	0.065	−1.680	0.048	0.952
Matrix metalloprotein	54	1.035	0.114	0.307	0.620	0.380
B. +UV, no supplements						
NQO1	15	1.447	0.192	2.330	0.982	0.018
xCT	15	0.779	0.096	−2.269	0.020	0.980
HO-1	15	0.875	0.100	−1.249	0.116	0.884
IL-6	15	22.765	5.068	4.294	1.000	0.000
IL-1β	15	2.262	0.401	3.146	0.997	0.004
TNF-α	15	0.768	0.130	−1.782	0.048	0.952
IL-17	15	0.573	0.075	−5.688	0.000	1.000
STING	15	0.953	0.088	−0.531	0.302	0.698
CYR61	15	1.763	0.211	3.626	0.999	0.001
MMP2	15	0.545	0.100	−4.557	0.000	1.000
MMP9	15	1.114	0.226	0.505	0.689	0.311
MMP3	15	8.871	2.655	2.964	0.995	0.005
Cytoprotective/antioxidant	54	4.279	1.478	2.552	0.993	0.007
Inflammatory	108	2.307	0.685	1.908	0.970	0.030
Matrix metalloprotein	18	7.565	2.311	2.841	0.995	0.006
C. +UV, +supplements						
NQO1	18	1.678	0.220	3.085	0.997	0.003
xCT	18	1.344	0.198	1.741	0.950	0.050
HO-1	18	1.122	0.134	0.905	0.811	0.189
IL-6	18	1.915	0.459	1.994	0.969	0.031
IL-1β	18	1.370	0.278	1.331	0.900	0.101
TNF-α	18	0.964	0.176	−0.202	0.421	0.579
IL-17	18	1.149	0.190	0.787	0.779	0.221
STING	18	1.022	0.114	0.195	0.576	0.4244
CYR61	18	1.077	0.153	0.504	0.690	0.310
MMP2	18	1.229	0.226	1.015	0.838	0.162
MMP9	18	1.409	0.193	2.121	0.976	0.025
MMP3	18	1.084	0.018	0.480	0.682	0.319
Cytoprotective/antioxidant	54	1.382	0.111	3.443	0.999	0.001
Inflammatory	108	1.250	0.107	2.344	0.990	0.010
Matrix metalloprotein	54	1.241	0.114	2.107	0.980	0.020

## Data Availability

The original contributions presented in this study are included in the article/Supplementary Materials. Further inquiries can be directed to the corresponding authors.

## Supplementary Materials

The following supporting information can be downloaded at: https://www.mdpi.com/article/10.3390/metabo15060360/s1, Figure S1: CONSORT diagram; Figure S2: DK/EPI index of number of dyskeratotic keratinocytes (DK) per epidermal area (EPI) in biopsies of subjects; Figure S3: Relative gene expression in peripheral blood mononuclear cells (PBMC) taken 1 day following a single UVB irradiation of subjects who had taken supplements. A. GR, B. CUR, C. GR + CUR. Each data point (*n* = 6 subjects per supplement treatment) represents a comparison (“fold-change”) to expression from PBMCs taken from the same individual 1 day following UVB irradiation in the non-intervention phase. Boxes envelop the 25th to 75th percentile of the data, whiskers are at the 5th and 95th percentile, and horizontal lines within boxes denote medians; Figure S4: Relative gene expression in skin-punch biopsies taken 1 day following a single UVB irradiation of subjects who *had* taken supplements (and compared to irradiated skin from the same individual). A. GR, B. GR + CUR, C. CUR. Each data point (*n* = 6 subjects per supplement treatment) represents a comparison (“fold-change”) to expression from a control biopsy taken from the same subject. Boxes envelop the 25th to 75th percentile of the data, whiskers are at the 5th and 95th percentile, and horizontal lines within boxes denote medians; Table S1: Primer sequences used for gene amplification; Table S2: Gene targets and their functions.

## Abbreviations

The following abbreviations are used in this manuscript:

AGE	advanced glycation endproduct(s)
AhR	aryl hydrocarbon receptor
AP1	activation protein-1
COX	cyclooxygenase
CUR	curcumin
DK	dyskeratotic keratinocytes
DTC	dithiocarbamate(s)
EPI	epidermal area
GR	glucoraphanin
IL	interleukin
Keap1	Kelch-like ECH-associated protein 1
LTB4	leukotriene B4
LTC4	leukotriene C4
M.E.D.	minimum erythematous dose
MMP	matrix metalloproteinase(s)
NF-кB	nuclear factor kappa B
Nrf2	nuclear factor-erythroid 2 p45-related factor 2
PBMC	peripheral blood mononuclear cells
PGE2	prostaglandin E synthase 2
SF	sulforaphane
TNFα	tumor necrosis factor alpha
UV	ultraviolet
VEGF	vascular endothelial growth factor
