# Supplementary material for: Oral Glucoraphanin and Curcumin Supplements Modulate Key Cytoprotective Enzymes in the Skin of Healthy Human Subjects: A Randomized Trial
Source: Metabolites. 2025 May 29;15(6):360. doi: 10.3390/metabo15060360 (PMC12195189; doi:10.3390/metabo15060360)
Supplement: Supplementary file 1 [file metabolites-15-00360-s001.zip › Supplementary Materials for Applied Sciences - April 30 2025.pdf]

Supplementary Figure S1. CONSORT Diagram.

Supplemental Figure S2. DK/EPI index of number of dyskeratotic keratinocytes (DK) per epidermal area (EPI) in biopsies of subjects.

Supplemental Figure S3. Relative gene expression in peripheral blood mononuclear cells (PBMC) taken 1 day following a single UVB irradiation of subjects who had taken supplements. **A.** GR, **B.** CUR, **C.** GR + CUR. Each data point (n=6 subjects per supplement treatment) represents a comparison ("fold-change") to expression from PBMCs taken from the same individual 1 day following UVB irradiation in the non-intervention phase. Boxes envelop the 25th to 75th percentile of the data, whiskers are at the 5th and 95th percentile, and horizontal lines within boxes denote medians.

Supplemental Figure S4. Relative gene expression in skin-punch biopsies taken 1 day following a single UVB irradiation of subjects who *had* taken supplements (and compared to irradiated skin from the same individual). **A.** GR, **B.** GR + CUR, **C.** CUR. Each data point (n=6 subjects per supplement treatment) represents a comparison ("fold-change") to expression from a control biopsy taken from the same subject. Boxes envelop the 25th to 75th percentile of the data, whiskers are at the 5th and 95th percentile, and horizontal lines within boxes denote medians.

Figure S1. CONSORT Flow Chart.

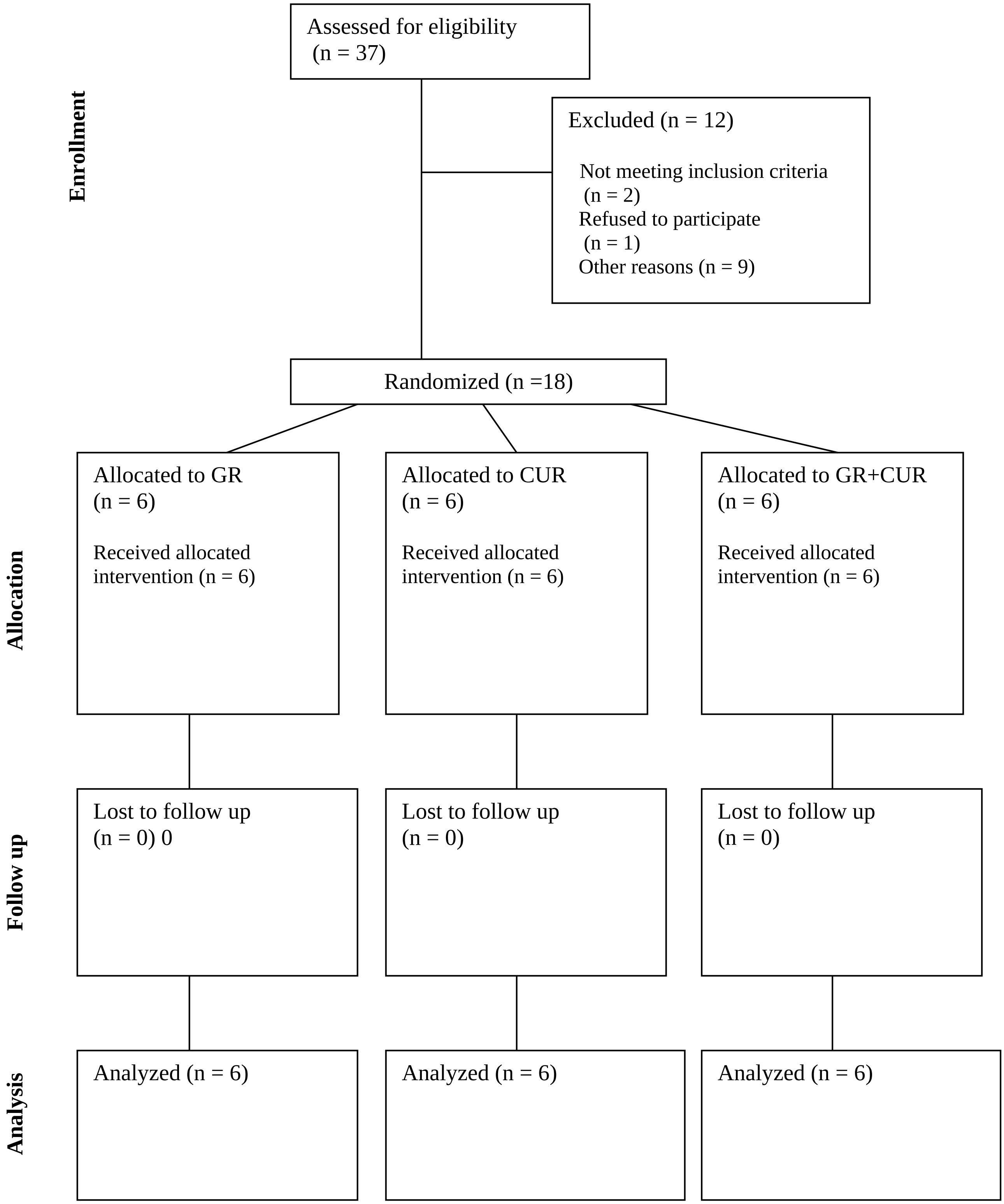

Supplemental Figure S2.

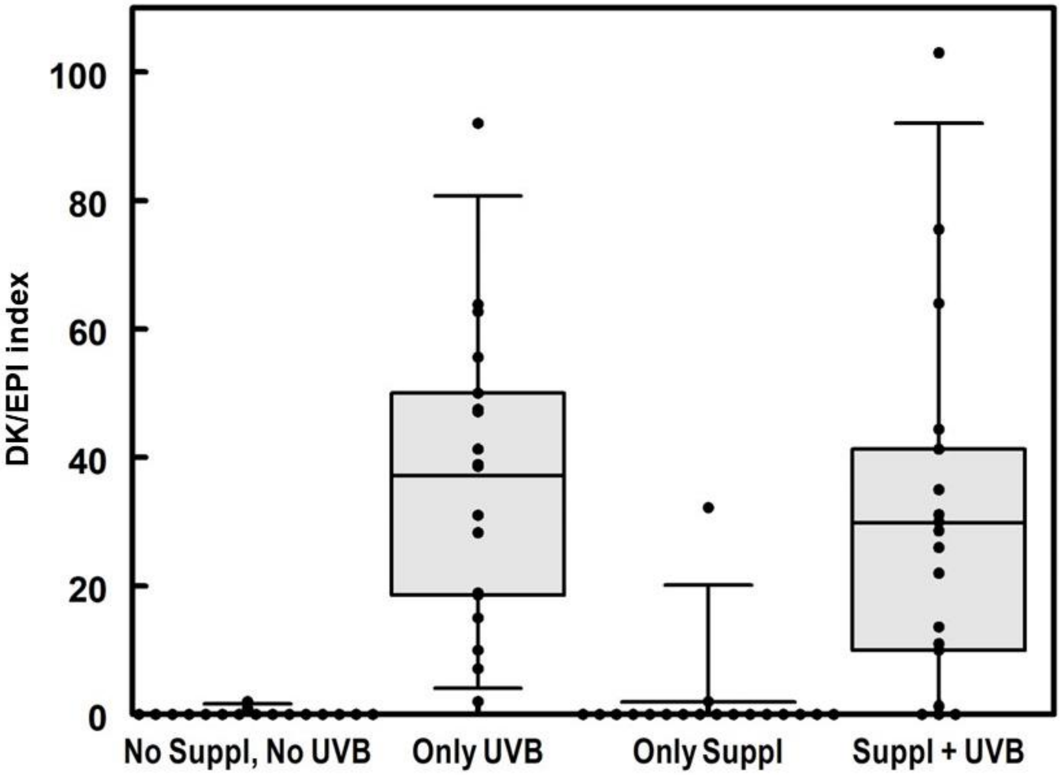

Supplemental Figure S3.

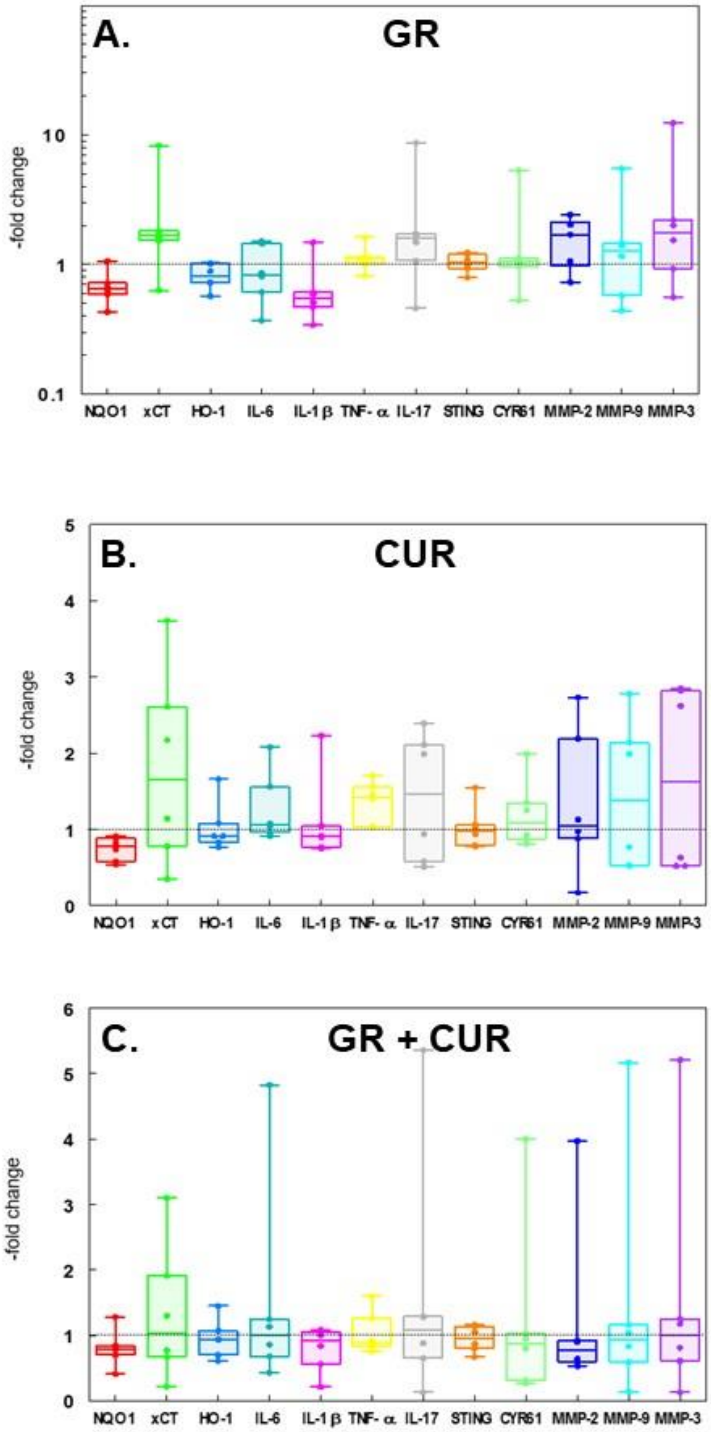

Supplemental Figure S4.

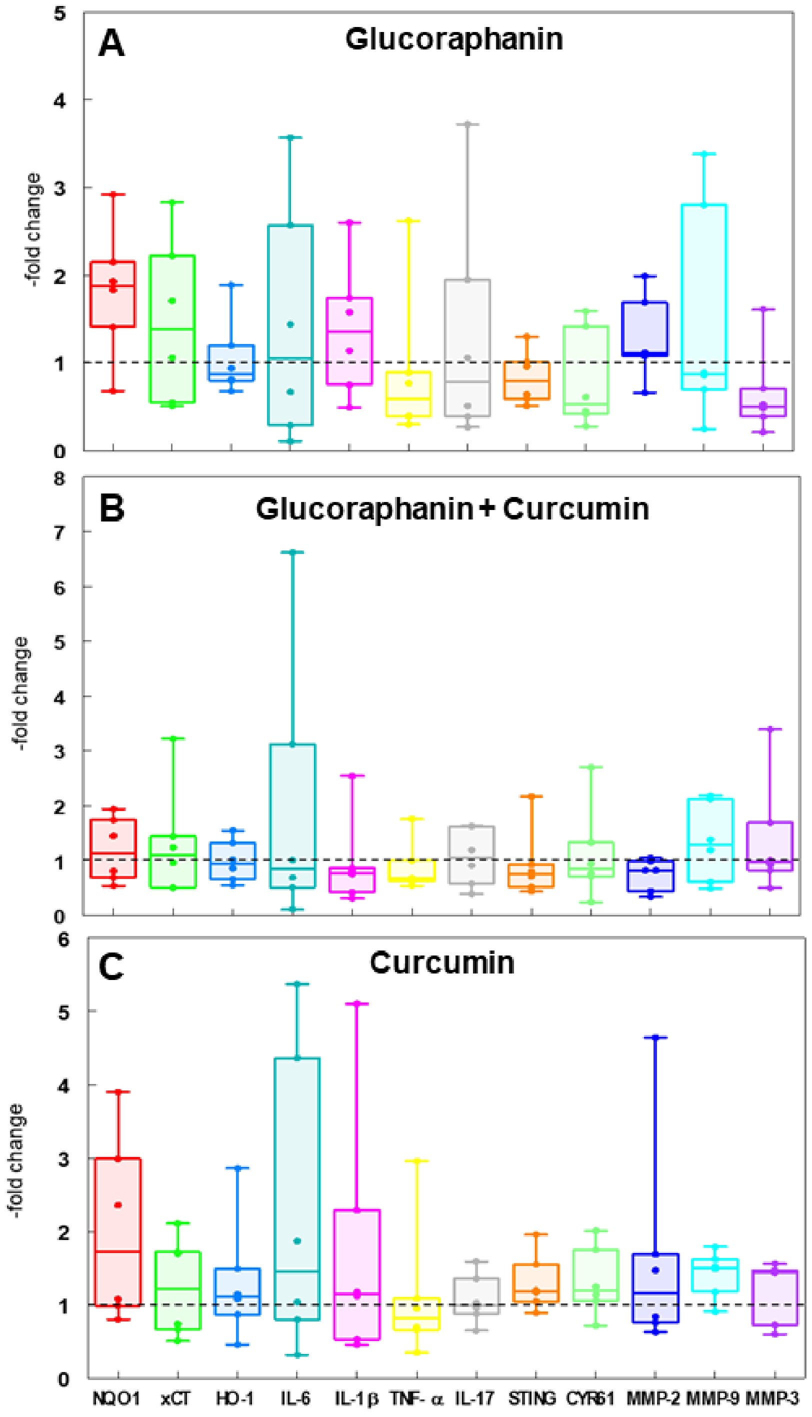

Supplemental Table S1.

Table S1. Sequences of real-time PCR primers.

Primers		Sequences
**NQO1**	forward	5'-CAG CTC ACC GAG AGC CTA GT-3'
	reverse	5'-GAG TGA GCC AGT ACG ATC AGT G-3'
**xCT**	forward	5'-GCC CAA GGG GAG ACA CAA AA-3'
	reverse	5'-TGT GCG ACT CAT AGA ATA ACT GC-3'
**HO-1**	forward	5'-GGG TGA TAG AAG AGG CCA AGA-3'
	reverse	5'-AGC TCC TGC AAC TCC TCA AA-3'
**IL-6**	forward	5'-CGA GCC CAC CGG GAA CGA AA-3'
	reverse	5'-GGA CCG AAG GCG CTT GTG GAG-3'
**IL-1β**	forward	5'-CAC GCT CCG GGA CTC ACA GC-3'
	reverse	5'-GGA GAA CAC CAC TTG TTG CTC CA-3'
**TNF-α**	forward	5'-ATC TTC TCG AAC CCC GAG TGA-3'
	reverse	5'-CGG TTC AGC CAC TGG AGC T-3'
**IL-17**	forward	5'-CTC TGT GAT CTG GGA GGC AA-3'
	reverse	5'-CAT GTG GTA GTC CAC GTT CC-3'
**STING**	forward	5'-CTT GGT TCT GCT GAG TGC CT-3'
	reverse	5'-CCG GTA CCT GGA GTG GAT GT-3'
**CYR61**	forward	5'-TCA AAG ACC TGT GGA ACT GGT ATC-3'
	reverse	5'-CAC AAA TCC GGG TTT CTT TCA-3'
**MMP2**	forward	5'-ATA ACC TGG ATG CCG TCG T-3'
	reverse	5'-AGG CAC CCT TGA AGA AGT AGC-3'
**MMP9**	forward	5'-CGG TGA TTG ACG ACG CCT TT-3'
	reverse	5'-ACC AAA CTG GAT GAC GAT GTC TG-3'
**MMP3**	forward	5'-GGA GTT CCT GAT GTT GGT CAC-3'
	reverse	5'-ATC TGG TGT ATA ATT CAC AAT CCT GTA-3'
**GAPDH**	forward	5'-TGG TAT CGT GGA AGG ACT CA-3'
	reverse	5'-GGG CCA TCG ACA GTC TTC-3'

Supplemental Table S2. Synonyms and functions of genes evaluated in skin punch biopsies as biomarkers.

Gene	Descriptive Name	Function
NQO1	(NAD(P)H Quinone Dehydrogenase 1)	Encodes a cytoplasmic 2-electron reductase; prevents on electron reduction of quinones that results in production of radical species
xCT	cystine-glutamate antiporter	implicated in supporting tumor growth and T-cell proliferation
HO-1 (HMOX-1)	heme oxygenase	essential enzyme in heme catabolism, cleaving heme to form biliverdin which then gets converted to bilirubin and carbon monoxide
IL-6	interleukin 6	a cytokine that functions in inflammation and the maturation of B-cells
IL-1β	interleukin 1β	a cytokine produced by activated macrophages as a proprotein; an important mediator of inflammatory response involved in cell proliferation, differentiation and apoptosis as well as in induction of COX2 in the CNS which contributes to inflammatory pain hypersensitivity
TNF-α	tumor necrosis factor α	a proinflammatory cytokine mainly secreted by macrophages; involved in regulation of cell proliferation, differentiation, apoptosis, lipid metabolism & coagulation; implicated in very many diseases
IL-17	interleukin 17	a proinflammatory cytokine produced by activated T-cells; high levels associated with several chronic inflammatory diseases; plays crucial role in host defense, cell trafficking, immune modulation, tissue repair and induction of innate immune defenses
STING	stimulator of interferon response CGAMP Interactor 1	encodes a pattern recognition receptor that plays a role in apoptotic signaling by associating with type II major histocompatibility complex
CYR61	CCN1 or cellular communication network factor 1	the secreted protein encoded by this gene is growth factor inducible and promotes the adhesion of endothelial cells; plays role in cell proliferation , differentiation, angiogenesis, apoptosis & extracellular matrix formation
MMP-2	matrix metalloproteinase 2	zinc-dependent enzymes capable of cleaving components of the extracelluar matrix & molecules involved in signal transduction; a gelatinase A, type IV collagenase
MMP-3	matrix metalloproteinase 3	zinc-dependent enzymes capable of cleaving components of the extracelluar matrix & molecules involved in signal transduction; a degrader of fibronectin, laminin, collages III, IV, IX & X, and cartilage proteoglycans

MMP-9	matrix metalloproteinase 9	zinc-dependent enzymes capable of cleaving components of the extracelluar matrix & molecules involved in signal transduction; a degrader of type IV and V collagens
